# Abstract of Paper on the Results of One Hundred and Forty-Seven Operations for Retroversion of the Uterus*Read before the American Gynecological Society at Washington, May 6th, 1897.

**Published:** 1897-07

**Authors:** A. Lapthorn Smith

**Affiliations:** B. A., M. D., M. R. C. S., England; Fellow of American Gynecological Society; Surgeon-in-Chief of Samaritan Free Hospital for Women; Gynecologist to the Western Hospital, and to the Montreal Dispensary; Professor of Clinical Gynecology in Bishops University, Montreal


### ABSTRACT OF PAPER ON THE RESULTS OF ONE HUNDRED AND FORTY-SEVEN OPERATIONS FOR RETROVERSION OF THE UTERUS.*

BY A. LAPTHORN SMITH, B. A., M. D., M. R. C. S., ENGLAND.

Fellow of American Gynecological Society; Surgeon-in-Chief of Samaritan Free Hospital for Women; Gynecologist to the Western Hospital, and to the Montreal Dispensary; Professor of Clinical Gynecology in Bishops University, Montreal.

This paper was based on ninety-four ventrofixations and fifty-three Alexander's operations. He held that ventrofixation was the only operation that should be entertained in cases of retroversion with adhesions; but it should not be done when the uterus was movable and when there was no disease of the appendages requiring abdominal section, in which cases Alexander's operation had given excellent results. There should be no death-rate to either operation, neither should there ever be hernia, either ventral or inguinal, if the following directions were followed. The two operations were equally easy, although a few years ago the author was opposed to Alexander's operation on account of its difficulty. Now he could invariably find the ligaments, and generally in from half a minute to a minute and a half. He warned his hearers not to do Alexander's operation if there were any adhesions, even if they were loose enough to permit the uterus to be lifted up, because they would be put upon the stretch and would drag so much upon the ligaments as to finally pull

* Read before the American Gynecological Society at Washington, May 6th, 1897.

them out of their anchorage. In laying down the technique of Alexander's operation, he placed great stress upon the importance of putting aside all cutting instruments as soon as the skin, superficial and deep fascia, had been cut through. Instead of laying open the inguinal canal as advocated by some writers, he advised his hearers not to cut a single fibre of the intercolumnar fascia which was the principal support of the pillars. Moreover, he said, the slightest nick of the fascia of the internal oblique would lead to a false passage and failure to find the ligament. If no cutting instruments were used, but only a Poeans forceps to draw out the ligament, there would be no difficulty in finding it, because there was nothing else in the canal but the ligament. In fact, with the eyes bandaged it could be found and drawn out, simply by introducing the closed forceps and then opening them, when the round ligament will fall into them and can be drawn out. He advocated the use of fine silk-worm gut which could be thoroughly sterilized and left in permanently. Occasionally he had been obliged to remove a buried stitch. In case any fibres of the intercolumnar or internal oblique should be accidentally cut, great care should be exercised in sewing them up to avoid hernia. He had only had one relapse after ventrofixation and one after Alexander, which were both subsequently repaired. Several of the cases of ventrofixation had since become pregnant and had had normal confinements. Also several cases of Alexander had had children. Many of the patients had been bed-ridden invalids for years before and were now enjoying excellent health. Both operations, each in its proper sphere, had given the greatest possible satisfaction.

